# Progesterone, Lipoic Acid, and Sulforaphane as Promising Antioxidants for Retinal Diseases: A Review

**DOI:** 10.3390/antiox8030053

**Published:** 2019-03-02

**Authors:** Vicente Hernández-Rabaza, Rosa López-Pedrajas, Inmaculada Almansa

**Affiliations:** Departamento Ciencias Biomédicas, Facultad de Ciencias de la Salud, Universidad Cardenal Herrera-CEU, CEU Universities, 46115 Valencia, Spain; vicente.hernandez@uchceu.es (V.H.-R.); rosa.lopez@uchceu.es (R.L.-P.)

**Keywords:** sulforaphane, progesterone, lipoic acid, retinitis pigmentosa, retinal diseases, antioxidants, reactive oxygen species, macular degeneration, diabetes retinopathy

## Abstract

Oxidative stress has been documented to be a key factor in the cause and progression of different retinal diseases. Oxidative cellular unbalance triggers a sequence of reactions which prompt cell degeneration and retinal dysfunction, both hallmarks of several retinal pathologies. There is no effective treatment, yet, for many retinal diseases. Antioxidant treatment have been pointed out to be an encouraging palliative treatment; the beneficial effects documented involve slowing the progression of the disease, a reduction of cell degeneration, and improvement of retinal functions. There is a vast information corpus on antioxidant candidates. In this review, we expose three of the main antioxidant treatments, selected for their promising results that has been reported to date. Recently, the sulforaphane, an isothiocyanate molecule, has been unveiled as a neuroprotective candidate, by its antioxidant properties. Progesterone, a neurosteroid has been proposed to be a solid and effective neuroprotective agent. Finally, the lipoic acid, an organosulfur compound, is a well-recognized antioxidant. All of them, have been tested and studied on different retinal disease models. In this review, we summarized the published results of these works, to offer a general view of the current antioxidant treatment advances, including the main effects and mechanisms described.

## 1. Introduction

Oxidative stress has been implicated in the pathogenesis of several eye diseases [1,2,3,4,5]. The retina is a tissue especially sensitive to oxidation, and is prone to generation of reactive oxygen species (ROS), due to the very high oxygen levels in the choroid, its high metabolic rates, and intense exposure to light [6,7,8]. Moreover, the retina has a high oxygen tension (70 mm Hg) which makes it very vulnerable to oxidative stress [9,10]. In the retina, the photoreceptors transduce the light into an electrical signal that is readable by the nervous system. In these transductor cells, ROS can be generated as a product of photochemical reactions, or as a result of cellular metabolism [11,12]. It has been described that the adenosine triphosphate (ATP) necessary for phototransduction is produced by the electron transport chain complexes in the outer segment, which is also a major source of reactive oxygen intermediates [13,14]. In addition, the outer segment is an area rich in polyunsaturated fatty acids, which means that this region is more sensitive to oxidation by ROS [15]. The focus on the outer segment is an updated topic, as because, traditionally, the inner segment of photoreceptor (which contains the mitochondria) has been considered to be a source of reactive oxygen intermediates, but Roehlecke et al. have described that ROS generation and oxidative stress occurs directly in the outer segment of photoreceptors [15].

Retinitis pigmentosa (RP), diabetes retinopathy and age-related macular degeneration (AMD) represent the causes of millions of blindness in the world. The term RP includes a large group of hereditary retinopathies, which are genetically and clinically heterogeneous. It is the most common cause of hereditary blindness [16]. Despite the diversity of retinal degeneration disorders, apoptosis of photoreceptors seems to be a feature common to all [17,18]. RP develops as a result of defects in genes responsible for upholding the structural or functional integrity of photoreceptors. In the most typical progression of RP, rods die first, due to mutation and this is followed by a mutation-independent cone cell death [2]. It seems that the survival of the cones depends on the rods and that once the rods die, the death of the cones is inevitable. This sequence of degeneration is related to the oxidative stress unbalance in retinal diseases. Due to the death or inactivity of rod photoreceptors (as it is known—the most abundant photoreceptor in the retina), oxidative stress might either be caused or exacerbated by reduced oxygen utilization, leading to outer retinal hyperoxia [19], which can induce ROS formation [20,21].

As in the RP, accumulating evidence has implicated oxidative stress as an important pathogenic factor in the AMD and the diabetes retinopathy [22,23,24,25]. It has been reported that diabetes retinopathy is accompanied by an increase in malondialdehyde (a product of lipid peroxidation), a decrease in glutathione (GSH) concentration, and decrease in glutathione peroxidase (GPx) activity [25]. Moreover, the activities of other antioxidant defense enzymes, such as superoxide dismutase (SOD), GSH reductase, and catalase, are diminished in the retina [26,27,28]. Photoreceptors could contribute to retinal pathology in diabetes, but how this could happen has not been demonstrated. Possible mechanisms include: (a) hypoxia resulting from high metabolism by photoreceptors; (b) excessive generation of ROS, perhaps from hyperglycemia-induced defects in mitochondrial electron transport; (c) altered metabolism or function of other neurons in the retina, secondary to abnormalities in the photoreceptors; or (d) defects caused by visual processes (phototransduction or visual cycle activity) within these specialized cells [29]. Finally, the AMD is produced by lesion of the macula, including photoreceptors, retinal pigment epithelium (RPE), and Bruch´s membrane damage [30,31]. AMD is the main cause of blindness in the developing world but the mechanism and causes of the AMD are not still well understood. Oxidative stress has been proposed to be a relevant factor in the etiopathology of the disease, in part, due to the phototoxic effect over the RPE cells, mainly by lipofuscin accumulation, and the activity decrease of the endogenous antioxidant system [22,23].

Oxidative stress is not the only factor involved in the development of the retinal dysfunction and degeneration, other factors, such as inflammation, vascular alterations, and cell degeneration, are also principal players implicated in the development and cause of retinal diseases. However, the reduction of oxidative stress has been unveiled to be an effective palliative treatment for several retinal diseases. Progesterone, lipoic acid (LA), and sulforaphane (SFN), have long been proposed to be antioxidant candidates, supported by a large group of result that remark on their healthy and therapeutic properties in several retinal disease models. To offer a general view that helps to evaluate their therapeutic potential, and to understand the shared properties of these molecules, we have reviewed the publications that discuss the roles of these molecules in retinal diseases, focusing specifically on the antioxidant properties and exclusively on the cellular and animal model data. The well-documented properties of these molecules, the number of publications related to RP, AMD, and diabetes retinopathy, and the expertise of the authors are the reason for reviewing these molecules, among other antioxidant candidates.

## 2. Progesterone as an Antioxidant in Retina Degeneration 

Neuroactive steroids or neurosteroids are steroid hormones synthesized within the central nervous system (CNS), from its precursor, cholesterol [32]. One of these neurosteroids, progesterone, is a C-21 steroid hormone and the biosynthesis of this hormone is a multistep process [33]. The retina is considered to be an authentic steroidogenic structure of the CNS, in which steroids synthesis is integrated in its circuits, for a conceivable role in the visual function [34]. The renewal process also involves RPE, where cholesterol is additionally provided from an extracellular source, through low density lipoprotein (LDL) receptors and the apolipoprotein E (ApoE), synthetized locally [35]. ApoE is also formed in retinal Müller glial cells or is internalized by ganglion cells, from which it can be rapidly transported into the optic nerve and the brain [35]. Recently, high cholesterol in RPE, changes in ApoE expression by Müller glia of the human retina, and the apolipoprotein polymorphism have been suggested to increase the risk of age-related macular degeneration, the impairment of visual function during aging, and the progression of glaucoma [35,36].

**Figure 1:**
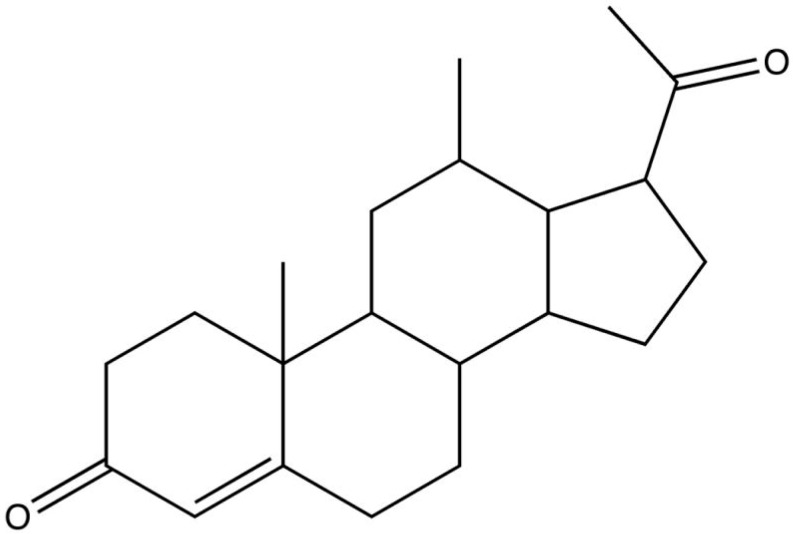
Chemical structure of progesterone [42].

As can be seen in Figure 1, progesterone does not have the characteristic chemical structure of an antioxidant, but high levels of the hormone appear to reduce free radical damage [37]. It is known that progesterone reduces the damage induced by free radicals [38]. As will be discussed later, there are studies that have shown that progesterone increases the expression of antioxidant enzymes, such as SOD [39]. All these studies suggest that progesterone reduces lipid peroxidation and oxidative stress, probably by reducing the generation of free radicals and intensifying the endogenous systems of neutralization of these radicals [40]. Particularly, in our research group, among other findings, we have also demonstrated that progesterone can decrease malondialdehyde concentrations in the retinal degeneration 1 (rd1) retina [41].

Nuclear progesterone receptor mRNAs have been identified in the retina and the choroid [43], while membrane-associated progesterone receptor component 1 (PGRMC1) has been identified in photoreceptor and Müller glial cells of the retina [44].

Regarding models of inherited retinal degeneration (rd), the literature strongly supports an antioxidant-based therapeutic approach in rd models [1,45] and in several instances, neurotrophic factors attenuate the photoreceptor degeneration [46,47], however, little is known about the effects of steroid sex hormones on disease progression.

Moreover, of the antioxidant properties, progesterone has multiple neuroprotective biological functions in the CNS, which interact and supplement with the antioxidant effects. It has been described that progesterone displays a protective role in different diseases, through the induction of cell survival and cell proliferation [48,49]. Progesterone acts at different levels to interrupt destructive processes. Some of its main actions include upregulation of the inhibition of gamma-Aminobutyric acid (GABA), decreasing lipid peroxidation and oxidative stress, reducing the release of inflammatory cytokines, and decreasing cell death by apoptosis [50].

### Oxidative Stress and Retinitis Pigmentosa: The Role of Progesterone

The use of antioxidants has gained importance as an alternative to treating RP. Several compounds have been studied as potential candidates for neuroprotective therapy with antioxidants, for RP. Among them, the most important ones are—tauroursodeoxicolic acid (TUDCA) [51]; curcumin [52]; mixtures of antioxidants, such as α-tocopherol, ascorbic acid and α-lipoic acid [2]; and N-acetylcysteine [53]. In addition, our research group has found that the combination of lutein, zeaxanthin, α-LA, and GSH, can delay degeneration in the rd1 mice [3]. A similar treatment increased the activity of GPx and GSH concentration and decreased cystine concentration in rd1 mice [1]. Other approaches have been used in studies aimed at the modulation of the endogenous antioxidant machinery. For example, some authors have demonstrated in a transgenic mouse model that the increased expression of type 4 GPx, protect the structure and function of photoreceptors [54]. Usui and colleagues have shown that in rd10 mice, the simultaneous increase of the expression of SOD 2 and catalase, in the same subcellular compartment (mitochondria), reduces superoxide radicals and oxidative damage in the retina [45], thus, improving the functionality of the cones and reducing cell death.

Studies have shown that the administration of Norgestrel (a synthetic progestin derivative) in two different RP mice models (light-induced degeneration and rd10 mice), leads to a decrease in photoreceptor apoptosis and improves electroretinogram (ERG) [55]. These authors proposed that the neuroprotective mechanism of progesterone is related to the growth factor basic fibroblast growth factor (bFGF) and to kinases regulated by extracellular signals extracellular signal–regulated kinases 1 and 2 (ERK1/2) [55].

Our research group have shown that oral administration of 100 mg/kg of progesterone, every 2 days, starting on post-natal day 7, in rd1 mice, significantly conserves the number of photoreceptors and causes a decrease in cell death [41]. Our study also highlighted the multiple benefits of progesterone as it was able to (i) reduce the typical gliosis of this degeneration, (ii) decrease the retinal glutamate concentration, and (iii) increase the GSH concentration. Similar results were found when the administration of progesterone was done in the rd10 mouse model [56]. These results are consistent with those found by Guarneri [34], who pointed out that high levels of glutamate (such as those found in the retina of the rd1 and rd10 mice) lead to an inadequate expression in the retina of the steroidogenic enzymes and an alteration in the production of neurosteroids.

On the other hand, Wyse Jackson et al. have described that norgestrel is able to inhibit cell death by apoptosis due to the activation of PGRMC1. This receptor is upregulated in, both, the degenerating and the fully degenerated mouse retina, versus the wild-type control mouse retina. The same effect is observed in the 661W photoreceptor cell line. Norgestrel produces a significant enhancement in nuclear PGRMC1, after 60 min of treatment, which has implications for an increased amount of PGRMC1-dependent transcriptional activity [57].

Other studies have showed that Norgestrel acts via upregulation of the neurotrophic factors bFGF and the leukemia inhibitory factor (LIF). LIF is a potent neurotrophic factor which, interestingly, has been shown to have antioxidant effects in the retina [58]. Byrne et al. have shown that light-damage caused a production of intracellular ROS in photoreceptor cells, which was prevented by pre-treatment with Norgestrel [59]. They showed that one of the routes through which Norgestrel performed its action is through the overexpression of Nuclear-factor-E2-related factor (Nrf2) and its effector protein SOD2.

In our laboratory, we investigated the role of progesterone on gliosis. The results showed a reduction in cell death and gliosis, with a statistically significant reduction in glutamate and a significant increase in reduced GSH [41]. In line with these results, with another animal model, Roche et al. also observed that Norgestrel reduces Müller cell gliosis [60]. In the same way, Allen et al. showed that in a retinal disease model, induced by middle cerebral artery occlusion, the progesterone administration resulted in reduced ERG deficits, reduced glial fibrillary acidic protein (GFAP), and reduced RGC death [61]. For more details about the antioxidant effects of progesterone in experimental models of retinal pathology, please see Table 1. 

## 3. Lipoic Acid: An Antioxidant and Anti-Inflammatory Molecule

**Figure 2:**
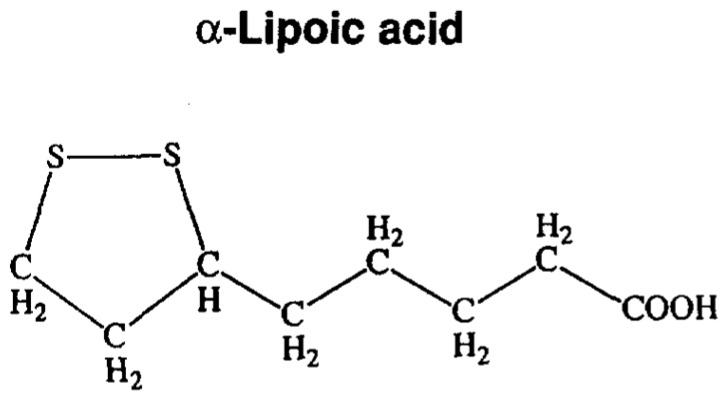
Chemical structure of α-Lipoic acid. Modified from [64].

LA, is a naturally dithiol compound synthesized enzymatically in the mitochondria from octanoic acid and a sulfur source [62,63]. The molecular structure of LA is represented in Figure 2. 

LA is a necessary cofactor for mitochondrial α-ketoacid dehydrogenases, and thus, serves a critical role in mitochondrial energy metabolism [63,65]. In the enzyme complexes, LA is linked by an amide bound to the γ-amino group of a lysine residue of the protein [66].

LA exists as two enantiomers—the R-enantiomer [R-LA or (+) LA] and the S-enantiomer [S-LA or (−) LA]. LA is present in the nature in R-form, but synthetic LA is a racemic mixture of R-enantiomer and S-enantiomer [66,67].

LA contains two thiol groups, which might be oxidized or reduced. In fact, LA is part of a redox pair, being the oxidized partner of the reduced form of the dihydrolipoic acid (DHLA). Moreover, both, the oxidized and reduced forms of LA have antioxidant capacity [66]. In fact, LA and DHLA were found to be highly reactive against a variety of ROS [68,69]. Therefore, LA is a potent reducing agent with the capacity to reduce the oxidized forms of several antioxidants, including vitamin C and GSH [66,70,71]. The regeneration of GSH is due to the capability of LA to recycle GSH from the Glutathione disulfide (GSSG; the oxidized form of GSH), and for its involvement in the GSH synthesis [67]. With regards to this, it is known that LA can increase nuclear Nrf2 levels, and this transcription factor regulates the γ-glutamylcysteine ligase (GCL) (the enzyme that controls GSH synthesis) [72,73]. In addition, the antioxidant capacity of LA might be due to the activity of the metal chelator.

It has also been described that LA and DHLA might have anti-inflammatory capacity. Several research studies have shown that LA inhibits the NF-κB translocation to the nucleus and its activation, and thus, the release of cytotoxic cytokines mediated by this transcription factor is attenuated [73,74]. In addition, ROS are critical intermediates for the NF-κB activating signals [75] and when it is activated, NF-κB promotes the expression of proteins that participate as central regulators of the immune, inflammatory, and apoptotic processes.

LA and DHLA are amphipathic molecules and might act as antioxidants and anti-inflammatory, both, in hydrophilic and lipophilic environments [69]. Therefore, it can induce its actions in, both, cytosol and plasma membrane of the cells [71].

LA is easily absorbed with the diet. Vegetables and animal tissues contain low amounts of R-LA. In animal tissues, LA is found in the kidney, the heart, and the liver. The most abundant plant sources of LA are spinach, followed by broccoli and tomatoes [69,76]. Moreover, several studies carried out in vivo, have shown that dietary supplementation with LA induced a decrease in oxidative stress, while restoring the diminished levels of the other antioxidants [71,77]. 

Due to the antioxidant and anti-inflammatory characteristics of LA, it has been reported that this compound protects against the damage of several pathologies, including neurodegenerative diseases [3,66,78]. This is also reinforced by the ability of the LA to cross the blood–brain barrier [71]. 

In addition to its antioxidant and anti-inflammatory properties, LA has been reported to increase tissue-sensitivity to glucose [79], probably by increasing the glucose uptake through the insulin-signaling cascade [80,81]. Studies on muscle cell lines have indicated that exposure to LA stimulates glucose uptake, by the redistribution of glucose transporters (GLUT1 and GLUT4) to the plasma membrane and further studies supports the role of insulin-mediated PI3K activity in LA-induced glucose uptake [63,67,80]. 

Due to these LA characteristics, there are numerous studies in which this molecule has been used as a treatment for diabetes in, both, experimental animals and in humans [81,82,83,84].

### 3.1. Diabetes Retinopathy and Lipoic Acid

In the next part, we will focus on the latest studies related to diabetic retinopathy and the effects of LA administration in experiments performed on laboratory animals.

As has been mentioned before, diabetes affects the retina microvasculature. Chen et al., investigated the effect of non-oral LA (intraperitoneal, intravitreous, and eye drops) on retinal capillaries in diabetic rats. Rats were evaluated using an ocular fluorometer to determine the penetration of fluorescein, through the blood–retinal barrier and microsections of retinal tissues were stained with hematoxylin and eosin, to study the blood vessel lesions. The researchers have demonstrated that capillary lesions in the retina of the diabetic rats were reduced by non-oral administration of LA, specifically, the administration form that obtained the best results was intraperitoneally administered LA [85]. Alvarez-Rivera et al. have studied a novel formulation of LA which is to be ocularly administered through eye drops, as a possible alternative to alleviate the effects of diabetes on cornea. Specifically, the researchers have developed an LA formulation based on micelles (Soluplus^®^, Ludwigshafen, Germany) [86]. They have shown that this kind of administration could be a good candidate, since the presence of LA was increased in experiments performed in bovine corneas [86].

LA can be administered as part of a dietary supplement; actually, there are several studies that have endorsed this form of LA administration, highlight, the results about the effects of a fortified extract over the retinal and plasma alterations in a streptozotocin-induced diabetic model [87]. This fortified extract contained—red berries, *Ginkgo biloba*, and white willow bark; among its components were carnosine and LA. In the diabetic group they have found an increase of TNF-α and vascular endothelial growth factor (VEGF) in the retina, and an increase of thiobarbituric acid reacting substances (compound used for measuring the peroxidation of fatty acids) in the plasma. Treatment with fortified extract significantly reduced retinal TNF-α and VEGF, and suppressed lipid peroxidation in the plasma. These results demonstrated that fortified extract might be useful in the treatment of diabetic retinopathy [87]. Other researchers administered dietary supplementation; including LA [84]. These researchers investigated the effect of this treatment on diabetic retinopathy induced in rats. Supplementation with the nutritional supplements, prevented the vascular pathology (capillary cell apoptosis, VEGF increase), the increase of inflammatory mediators (interleukin-1β and NF-κB), and total ROS levels. Moreover, this supplementation prevented the impairment of DNA mitochondria and alterations in the ERG. This study proposed that nutritional supplementation studied by researchers, might be a good candidate to improve diabetic retinopathy [84].

These results are in consonance with other studies. LA administrated orally in diabetic rats suppressed superoxide formation and prevented the change of nicotinamide adenine dinucleotide phosphate oxidase (NADPH oxidase) (they were involved in the production of oxidative stress). This antioxidant also reduced the expression of VEGF and other proteins related with angiogenesis (erythropoietin and angiopoietin 2), induced by diabetes. This study concluded that LA acting as an antioxidant could have an antiangiogenic effect and have beneficial and protective effects of diabetic retinopathy [88]. Kim et al., studied the effects of LA administered orally in diabetic mice and demonstrated that LA protected against the injured retinas of diabetic mice, by reducing oxidative stress [89]. Therefore, LA activated the adenosine monophosphate-activated (AMP-activated) protein kinase and inhibited the O-linked β-N-acetylglucasamine transferase. It is known that AMP-activated protein kinase activation has anti-apoptotic effects [90], this can be corroborated, as they have shown lowered levels of the pro-apoptotic marker cleaved caspase-3 in the diabetic retinal ganglion cells, after LA administration. Similar results have been shown after metformin treatment of retinal pigment epithelial cells exposed to high glucose [89]. It has been studied the effect of LA on the retinal ganglional cell, in diabetic mice [91]. Moreover, they investigated the thicknesses of various retinal layers and VEGF levels. The thicknesses of the full-length retina, outer nuclear layer (ONL), and inner nuclear layer (INL) were significantly reduced in the diabetic group, compared to the control and LA treatment groups. The number of ganglional cells in the diabetic group was lower and the VEGF expression was significantly higher than the control and the LA treatment groups. These results showed that LA treatment could protect the damage in the mouse diabetic retina, reducing VEGF levels, protecting ganglional cells, and preserving the thicknesses of the INL and the ONL. The researchers concluded that LA could be a candidate as a therapeutic supplement to decrease the injury on diabetic retinopathy [91]. Please see Table 2.

### 3.2. Retinitis Pigmentosa and Lipoic Acid

As already has been indicated in previous sections, RP is a group of diseases in which a mutation results in the death of photoreceptors, first the rods and then the death of cones takes place, progressively. Several theories have determined that the increase of oxidative stress induced by the death of the rods might be implied in the death of the cones photoreceptors [92]. It is widely known that photoreceptors death is mediated by apoptosis and the ROS might act as mediators of retinal cell apoptosis [93].

Due to the antioxidant capacity of LA, which has been argued previously, it could be a candidate for preventing the photoreceptors death. In fact, a variety of antioxidants has been tested as a treatment for RP in, both, animal [94,95,96] and human models [97,98].

Several investigations have studied ROS implication in the death of cones photoreceptors and the effect of LA administration as a treatment in a RP models. Komeima et al., [2]. administered a mixture of antioxidants (including LA, among others) in rd1 mice, a model of RP. After the antioxidant mixture administration, retinas of rd1 mice showed a reduction of the oxidative stress markers studied and this was associated with an increase in the cones density, in the rd1 retina, indicating an increase in the cones survival. The mixture of antioxidants also improved the retina function of rd1 mice observed through ERG [2]. The same group of researchers also studied the effect of the mixture of antioxidants in other models of RP—rd10 mice, a model of more slowly progressive RP, and Q344ter mice, a model of rapidly progressive RP. This antioxidant preserved the cone density and the retinal function, like what happens in mice with a faster progression in the death of photoreceptors, rd1. Therefore, in the Q344ter model, the cone density has been preserved too, after the administration of the antioxidant mixture [99]. The authors concluded that combining antioxidant therapy with other strategies might provide meaningful benefits to patients with RP. 

Studies performed in our research group have tested the effect of a mixture of antioxidants, (including LA) in an rd1 model, in vivo and in vitro. The antioxidant treatment rescued the number of rows and decreased the avidin (used to identify oxidatively damaged DNA) and terminal deoxynucleotidyl transferase dUTP (Deoxyuridine Triphosphate) nick end-labeling (TUNEL) positive cells, in the ONL, in rd1 retina explant. Similar effect has been found in retinas from animals, after the administration of the antioxidant mixture. This treatment could slow down the progression of photoreceptor death, by counteracting the oxidative stress. The antioxidants did not revert the RP disease but could delay the degeneration of the photoreceptors [3]. Markers of oxidative stress and endogenous antioxidant defenses were also measured in the retinas of the rd1 model. An increase in GPx activity and GSH concentration with a decrease in the cysteine concentration was found in the retinas of rd1 animals treated with the antioxidant combination. However, malondialdehyde levels (a product of lipid peroxidation) did not decrease significantly. The antioxidant mixture had also decreased the percentage of TUNEL and avidin positive cells in the retina of rd1 mice. This study had demonstrated the importance of maintaining thiol homeostasis, to protect against retinal cell death, since active GSH synthesis seems necessary to allow the antioxidant mixture to exert a sort of GSH-sparing effect on the retinas of the treated animals. This GSH effect has been demonstrated through experiments with buthionine sulfoximine treatment (an inhibitor of γ-glutamylcysteine synthetase, the enzyme that controls the GSH synthesis) [1]. When LA is administered in combination with progesterone in rd1 mice, Ramirez-Lamelas et al., [5] have demonstrated a decrease in photoreceptors death, and in the GFAP staining; the overexpression of GFAP can be used as an indicator of stress and retinal damage, as well as the activation of Müller cells in rd1 mice retinas [5]. These results highlight that progesterone and LA administered in combination have better results than when administered separately [5]. To more details about the treatments, please see Table 2.

## 4. Sulforaphane as Antioxidant Treatment in Retinal Diseases

### 4.1. Sulforaphane Overview

**Figure 3:**
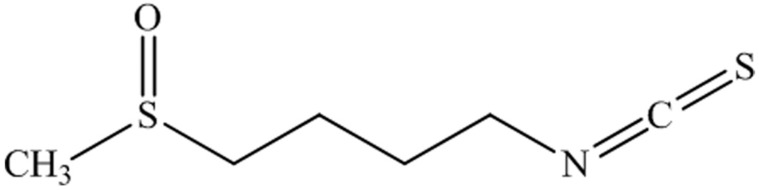
Chemical structure of the sulforaphane compound showing the nature of sulfur. Modified from [117].

SFN is an isothiocyanate molecule, as may be appreciated in the chemical structure (Figure 3), present in cruciferous vegetables, broccoli being the most relevant example of a natural SFN source [100]. During the last few decades, SFN has been revealed to be a neuroprotective candidate because of its antioxidant and anti-inflammatory effects [101,102,103]. The SFN induces the expression of the phase 2 genes, through activation of the Nrf2. These genes encode for different antioxidant enzymes, including glutathione transferases or NAD(P)H dehydrogenase quinone [100]. The mechanism of action of SFN is based on the dissociation of the transcription factor Nrf2 of Keap1, a cytosolic repressor, promoting its translocation to the nucleus and inducing an antioxidant response. The binding of Nrf2 to the DNA promoter region antioxidant-responsive element (ARE), triggers the nuclear antioxidant response, through an increased expression and activity of reductive systems [104,105].

Based on its antioxidant properties, there is a recent and homogeneous list of publications point out the neuroprotective potential of SFN in several models of retinal diseases. In this literature review, we have summarized the SFN antioxidant effects and the mechanisms described on different retinal disease models, including animal and cultured cell models. Our goal is to show a general picture of the current SFN treatments advances, which focus especially on the effects and mechanism reported to date.

### 4.2. Age Macular Degeneration

RPE cells (RPE) protect the photoreceptors cells of the degeneration triggered by oxidative unbalance, which is produced by an increase in the levels of different ROS. The progressive dysfunction of the retinal epithelial cell layer is considered to be a hallmark of age macular degeneration [22,23]. To prevent retinal degeneration, the improvement of the antioxidant function of the retina layers seems to be a good strategy [106].

In 2001 and 2004 [107,108], two articles have demonstrated the antioxidant potential of the SFN over human RPE cells exposed to oxidant agents, including different chemical oxidative stressors and light exposition. The authors [107] analyzed the SFN effect on RPE cells exposed to four oxidative stressors (menadione, tert-butyl hydroperoxide, 4-hydroxynonenal, and peroxynitrite); the results indicated that the SFN reduced the cell death ratio, which was increased by the exposition to oxidative agents. Interestingly, the positive SFN effects were concentration-dependent and persistent, after the end of the treatment. Following this research, in a new publication, the authors demonstrated similar beneficial effects against the exposition to UV light, in the presence of all-trans-retinaldehyde [108]. In both works, the induction of the phase 2 genes was linked with the neuroprotective SFN effects. In relation to the antioxidant molecules, the authors showed a high correlation between the SFN concentrations and GSH levels. Regarding the study of the SFN antioxidant mechanisms, the authors used a genetically modified fibroblast, with different Keap1/Nrf2 complex alterations; the results indicated that the Nrf2 activation is crucial to the antioxidant effect induced by SFN through the phase 2 enzymes. For more details on these treatments, please see Table 3.

A year later, a new work was published on the effects of SFN on the Thioredoxin (Trx) system [109]. The Trx system is involved in the regulation of the internal redox state. In this work, the authors combined both, in vivo and in vitro studies. Using a human RPE cells cultured model (K-1034), the authors analyzed the SFN antioxidant mechanism. The results indicated that SFN induces Trx protein expression through the activation of the ARE, by binding Nrf2, (note that, other transcription factors were also detected to bind to ARE, such as the small Maf and c-jun). The study was complemented with the first in vivo results on SFN’s effects on retinal diseases; the authors explored the effects of the SFN against light-exposition damage. The exposition to white light (2 h) produces an increasing of TUNEL-positive cells (apoptotic marker), in the outer nuclear layer and the retinal pigment epithelial layer, the SFN reverted this cytotoxic effect. Moreover, SFN recovered retinal functional detriment, the ERG records indicated an improvement of the retinal function measured 96 h after light exposure. Note that the authors used two different types of SFN treatment, oral and intraperitoneal (i.p.), achieving the best neuroprotective results through the i.p. administration. For more details on these treatments, please see Table 3.

Reinforcing the previous results, in 2006 and 2008, two studies indicated the antioxidant effects of SFN on the RPE cells. In 2006 [110], a study showed that SFN protected the RPE cells against oxidative damage and, in parallel, produced an increase of GSH and GSH-S-transferases, as well as an increase in the activity and gene expression of the NAD(P)H:quinone reductase. A novelty method was presented in 2008 [111] to measure the redox state of the cells, using a ratio of reduced nicotinamide nucleotides NAD(P)H and oxidized flavoproteins (assayed by microscopic autofluorescence spectroscopy). The results indicated that SFN treatment increased the redox ratio and cell viability against different concentrations of hydrogen peroxide and tert-butyl hydroperoxide, the two oxidant agents used in the study. For more details on these treatments, please see Table 3.

To that time, different studies supported the contribution of the SFN to the cell viability of human RPE cells; the following studies were designed to get a deeper look into the SFN antioxidant mechanism. The next results reviewed were published in 2013 [112], the authors used the microarray technique to analyze the gene expression on the ARPE-19 cell line, 69 genes were altered by 6/12 h of SFN treatment, and the altered expression of 8 of them was confirmed by RT-PCR. From these 8 genes, four were upregulated by SFN, including NAD(P)H:quinone oxidoreductase (NQO1), Sulfiredoxin 1 (Srxn1), the modulatory subunit of the glutamate-cysteine ligase (GCLM), and Thioredoxin 1 (Trx1), all of which were involved in reduction and detoxification responses. The Thioredoxin interacting protein (Txnip) which has been demonstrated to inhibit Trx1 was downregulated when treated with SFN, together with the upregulated antioxidant genes, the results indicated that the redox reduction system was highly activated by SFN. The expression of Nrf2 was not upregulated but it was increased in the Nrf2 nuclear translocation, measured by nuclear extractions, suggesting that the SFN activation of the Nrf2/ARE system was achieved by translocation induction. The other genes analyzed by PCR were related to the inflammation response. Three years later, in 2016 [113], an in vivo research on retinal photooxidation showed a set of results which confirmed the increased expression of Nrf2 and Trx1 by SFN, but also, showed that the anti-caspase activity was added to the antioxidant activity elicited by SFN. For more details on these treatments, please see Table 3.

Interestingly, one year later [114], a published study, indicated a cytoprotective effect induced by SFN on the RPE-1 cells, but independent of the Keap-Nrf2-ARE pathway. The SFN increased the mitochondrial fusion, an inhibitor apoptosis mechanism, against an apoptotic inducer. However, the authors reported that this cytoprotective effect was independent of the Keap-Nrf2-ARE pathway. Using cells with Nrf2 genes depleted by the clustered regularly interspaced short palindromic repeats (CRISPR)/Cas9 method, the SFN treatment maintained its cytoprotective effect, due to the reduction of the fission/apoptotic factor Drp1. Finally, the last publication reviewed using human RPE cells was in 2018, this study [115] carried out an extensive analysis of the SFN effects against a hydrogen peroxide treatment, including cell viability, gene expression, and metabolic assessment alterations. The results supported the idea that the SFN protects the RPE cells viability against redox unbalances; through the induction of phase 2 enzymes, the response was concentration-dependent like that reported in other works. Additionally, the SFN treatment produced metabolic alterations, however, these metabolic changes were conditioned to the hydrogen peroxide presence, suggesting that the antioxidant response seemed to be conditioned to a previous oxidative state, and this conditioned respond was reported in previous works. Supporting this idea, in 2014 [116], a study evaluated the influence of different compounds in the storage of RPE cells (in one environment, without oxidative unbalance). The SFN did not demonstrate any effect over the survival of the cells; even the highest concentration used (50 mM) reduced the cell survival rate. For more details on these treatments, please see Table 3.

### 4.3. Retinitis Pigmentosa

In 2017, the SFN effects in a model of RP, rd10 mice was described [118]. The authors carried out a functional and cellular analysis of the control mice and the rd10 mice, treated with a vehicle or SFN. The SFN treatment improved the retinal function, reflected by the electroretinography results, including a higher a-wave and b-wave amplitudes triggered by SFN. The morphological analyses showed a reduction of the retinal cell degeneration after the SFN treatment, indicating a cytoprotective effect. For more details on these treatments, please see Table 3.

### 4.4. Diabetic Retinopathy

Inflammation or reduction of pericytes by advanced glycation end products (AGE) seems to contribute to diabetic retinopathy [119,120,121]. The AGE effects on pericytes are mediated by the interaction of AGE with an AGE receptor (RAGE). In 2014 [122], a publication indicated that SFN reduces the AGE effects on pericytes, by its antioxidative effects, in part through a suppression of the RAGE expression. This study showed, for the first time, an association between SFN and diabetic retinopathy. For more details on these treatments, please see Table 3.

### 4.5. Retinal Ischemia

In 2014 [123], a neuroprotective effect of SFN was reported in a rat retinal ischemia-reperfusion model; this animal model was induced by increased intraocular pressure. Morphological and molecular assessments showed a clear cytotoxic effect in the retina of this experimental model, including apoptosis of ganglion and amacrine cells, reduction of the inner retinal layer, and an increase in ROS and inflammatory mediators. All these effects were reverted by the SFN treatment. The authors described an increase of the Nrf2 and Heme oxygenase-1 (HO-1) expression through an SFN treatment. To analyze the contribution of HO-1 on the neuroprotective SFN effects, the authors administrated a selective HO-1 inhibitor that induced a reversion of the protective SFN effect, indicating the implication of HO-1 in the neuroprotective effects cited.

Supporting previous results, in 2015 [124], a new work was published, in which the authors administered SFN to a mice model of retinal ischemia-reperfusion, but this time the SFN concentration and time administration were found to be higher and shorter, respectively. The morphological retinal alterations, thinning of the inner retinal layer, and the dysfunction activity of the retina, measured by scotopic electroretinography, were reverted by SFN, specifically, the a and b waves amplitudes showed in a significant reduction in the control mice and was slightly reverted by the SFN. For more details on these treatments, please see Table 3.

### 4.6. Usher Syndrome, Tubby Model Mice

The tubby model mice expressed a photoreceptor degeneration, one of the alterations that characterize this model. In 2007, a study published showed that SFN affects retinal degeneration in this animal model [125]. The molecular PCR and morphological results, of this interesting work, indicated that the Trx and the TrxR (reductase) levels were reduced, prior to photoreceptor degeneration, indicating the initial and previous role of the oxidative unbalance on cellular death. This depletion of the endogenous redox system was reverted by the SFN treatment. Moreover, the SFN effects seemed to be regulated by external kinases signals, because the SFN effect was blocked by the inhibition of the kinases signal, through the inhibitor PD98059. These results suggest that the SFN activated the Nrf2, mediated by ERKs external signals. For more details on these treatments, please see Table 3.

## 5. Conclusions

Cellular oxidation is a key factor in the development of several retinal diseases. Antioxidant treatments have demonstrated the ability to slow retinal disease progression and improve retinal function. In the last decades, several candidates have been revealed as promising antioxidant treatments. In this review, we analyzed three of these candidates, progesterone, lipoic acid, and sulforaphane. The data reviewed on several retinal diseases, mostly point out that these antioxidant candidates display a neuroprotective potential, at the cell and functional level. To know more details about the treatments results, please see Table 1, Table 2 and Table 3.

Progesterone has been mainly tested in RP animal models, in general; all results indicated that oxidative stress plays a relevant role in the progression of the disease and that progesterone reduces cell degeneration and retinal dysfunction. The healthy effect seems to be produced by the induction of antioxidant molecules, such as GSH GPx and SOD2, and by the consequent reduction of oxidant molecules, such as superoxide radicals and Glutamate. The mechanism proposal includes the activation of the Nrf2 transcription factor pathway, probably by previous activation of external kinases, the induction of neurotrophic factors expression or the activation of progesterone receptors (PGRMC1). Along with the data from RP animal models, studies on ocular ischemia have also indicated a neuroprotective effect of progesterone.

Among the reviewed molecules, the lipoic acid is possibly the most and long-tested compound. It has documented the antioxidant and neuroprotective effects in RP and diabetic retinopathy animal models, as well as in clinical trials, with positive [126,127,128] and negative results [129]. Like progesterone, the LA reduces the progression of the cellular degeneration and improves the retinal function. Different studies have explored different administration vies, such as oral and no oral administration; the i.p. administration resulted in the most efficient administration but not the oral method. The LA nutritional dietary, as a supplementation treatment, has been proposed as a treatment in diabetic retinopathy, based on its antioxidant results, as well as anti-inflammatory and vascular effects. With regards to RP, highlighting the antioxidant combination studies of LA with another antioxidant, including progesterone, the results indicated an enhanced protection against retinal degeneration and improvement of retinal function, by a combination versus single administration. These results suggest that the specific effects of each antioxidant are different and that the best treatment would be achieved by an antioxidant mixture, for example progesterone seems to reduce malondialdehyde more efficiently than LA, but progesterone seems to produce a better cellular protection of retinal cells, when administrated with LA, probably by stronger anti-inflammatory and anti-apoptotic mechanisms. Future studies are necessary to unveil the potential of antioxidant mixtures. Regarding the mechanism, the LA studies supported the idea that the transcription induction of Nrf2 is the molecular pathway where the focus of future research should be.

We found more studies on SFN for the RPE cells degeneration models, related to age macular degeneration. These studies indicated that the SFN is a solid candidate as a neuroprotective agent against human RPE cells degeneration. The effects documented, describe the protection of the SFN against photooxidation and other oxidant agents, having an antioxidant potential, depending on the SFN concentration but being perdurable after the end of the treatment. The most tested cell-cultured model of RPE cell is the ARPE-19 cell model. Regarding the mechanisms proposed in the induction of phase 2 enzymes and the activation of the Trx system, the SFN treatments were correlated with gene expression of reductase GSH, GSH-S-Transferase, and NQO1. Concerning the transcription factor Nrf2, the SFN seems to induce a translocation to the nucleus more than increasing the expression.

Beyond the macular degeneration, the SFN has been analyzed in other retinal disease models, including RP, diabetes retinopathy, retinal ischemia, and one Usher animal model. In all studies reviewed, the SFN treatment was reported to revert the retinal dysfunction and the cell degeneration. This group of results clearly showed the antioxidant potential of the SFN. Moreover, concerning the antioxidant mechanisms, new approaches were added. As was expected, the results suggest a role of the SFN/Nrf2 pathway in bursting the Trx system and in the induction of the phase 2 genes, highlighting the HO-1 expression. However, the last dataset brings new ideas, such as the role of different kinases in the SFN/Nrf2 activation and anti-caspase pathways independent of the Nrf2 pathway, this research lines requires future analysis.

The anti-apoptotic and anti-inflammatory effects of the progesterone, LA, and SFN has been documented in different pathologies. However, based on the idea that the redox unbalances are the beginning of a greater pathological chain reaction, meaning, oxidative stress is followed by inflammation and posterior cellular degeneration, in this review we decided to focus on exploring the recent results about the first step—the oxidative alterations. However, the neuroprotective validation of the three compounds requires a future general analysis, including a future comparative review of the antioxidant, anti-inflammation, and cell viability properties.

Although they share an action mechanism, including the activation of the transcription factor Nrf2, or their anti-inflammatory properties, it is not possible to select one of the candidates among all, because there are no comparative studies, and it was not the objective of this review. Moreover, on viewing the updated results [130,131], other antioxidant candidates were found, such as polyphenolic phytochemicals, which deserves be considered for future investigations. Especially relevant are the current data of the Panfoli, I., research group [132], in relation to the molecular alterations of the outer segment and the related antioxidant mechanisms.

All results reviewed followed one common direction—the progesterone, the LA, and the SFN seemed to be good antioxidant candidates to palliate the degeneration and dysfunction of several retinal diseases. The recent results strongly support these antioxidants as neuroprotective molecules in different retinal diseases. Future research exploring the dose/respond, antioxidant combination effects, underlying mechanisms and molecular target of these compounds are necessary, as well as their roles in inflammation and cellular degeneration, but the current results are hopeful.

## Figures and Tables

**Table 1:** Antioxidant effects of progesterone in experimental models of retinal pathology.

Antioxidant: Progesterone		
**Retinitis Pigmentosa**		
**Authors**	**Study Type and Treatments**	**Results**
Doonan, F. et al., 2011. [55]	The light damage model and the rd10 model. Treatment: 100 mg/kg Norgestrel, i.p. Treatment was on alternate days commencing at P18.	Decreased photoreceptors apoptosis and improved electroretinogram
Sánchez-Vallejo, V. et al. 2015. [41]	rd1 model Treatment: 100 mg/kg progesterone, o.a. Treatment was on alternate days starting at P7.	Decreased cell death Reduced glial reaction Decreased oxidative stress
Wyse-Jackson, A.C. et al. 2016. [57]	Retina explants and photoreceptor derived 661W cell line. Treatment: 20 µM Norgestrel in vitro studies.	Increased in the quantity of PGRMC1. Cytoprotective effect
Byrne, A.M. et al. 2016. [59]	Light damage model Treatment:100 mg/kg Norgestrel, i.p	Rescue of photoreceptor cells from light-induced ROS production and cell death. Reduced morphological damage. Increased expression of Nrf2 and SOD2
Roche, S.L. et al. 2017 [60]	rd10 model Treatment: Norgestrel was added to the chow at a concentration of 0.05% (500 ppm).	Decreased microglial activity and Müller cell gliosis
Benlloch-Navarro, S. et al. 2019 [56]	rd10 model Treatment: 100 mg/kg progesterone, o.a.	Decreased retinal malondialdehyde, a lipid peroxidation product. Decreased photoreceptors cell death and reactive gliosis.
**Ocular Ischemia**		
**Authors**	**Study Type and Treatments**	**Results**
Allen, R.S. et al. 2015. [61]	Middle cerebral artery occlusion model. Treatment: 8 mg/kg progesterone i.p. at 1 h post injury, and then s.c. at 6, 24, and 48 h.	Reduced ERG deficits, upregulation of glutamine synthetase and GFAP, and cytoprotection (retinal ganglion).

ERG: electroretinography. GFAP: Glial fibrillary acidic protein. GSH: Glutathione. i.p.: Intraperitoneal injection. o.a.: Oral administration. PGRMC1: membrane-associated progesterone receptor component 1. ROS: Reactive oxygen species. s.c.: subcutaneous administration. SOD: Superoxide dismutase.

**Table 2:** Antioxidant effects of lipoic acid in experimental models of retinal pathology.

Antioxidant: Lipoic Acid		
**Diabetes Retinopathy**		
**Authors**	**Study Type and Treatments**	**Results**
Lee, S.G., et al., 2012. [88]	Streptozotocin-induced diabetic rats LA Treatment: 10 mg/kg o.a.	LA oral: Suppresses superoxide formation and increases NADPH oxidase. Reduces expression of VEGF, erythropoietin and angiopoietin 2.
Chen, C.L., et al., 2013. [85]	Streptozotocin-induced diabetic rats Treatment: 30 mg/kg/day LA i.p. 1% LA eyedrop 1% LA 1% HA intravitreal 1% LA 3% HA intravitreal	Decrease in fluorescein leakage from ocular vascular vessels and decrease in vascular lesion after LA administration (i.p.).
Bucolo, C. et al., 2013. [87]	Streptozotocin-induced diabetic rats LA Treatment: Fortified extract—containing LA (300mg/kg) among others (i.p.)	Treatment with fortified extract reduces retinal TNF-α and VEGF and suppresses lipid peroxidation in the plasma, in diabetic rats.
Kowrulu, R.A., et al., 2014. [84]	Streptozotocin-induced diabetic rats LA Treatment: Nutritional supplement—each kilogram contains 750 mg among others	Treatment with nutritional supplement: Decreases cell death, decreases VEGF and anti-inflammatory respond. Increases mitochondrial DNA-encoded proteins. Decreases total ROS levels. Prevents ERG alterations induced in diabetic rats.
Alvarez-Rivera, F., et al., 2016. [86]	LA ≥ 99% Soluplus^®^ Bovine corneas	LA with Soluplus ^®^ (Ludwigshafen, Germany) increases the LA presence in the bovine cornea.
Kan, E., et al., 2017. [91]	Streptozotocin-induced diabetic mice (BALB/C) LA Treatment: 100 mg/kg i.p.	LA i.p.: Increases the thicknesses of total retina, ONL, and inner nuclear layer; the number of retinal ganglion cells and decreases the VEGF expression in diabetic retinas.
Kim, Y.S., et al., 2018. [89]	Streptozotocin-induced diabetic mice LA Treatment: 200mg/kg o.a.	LA oral: Decreases 4-hydroxynonenal and increases glutathione peroxidase. Reverses the decreases activation AMP-activated protein kinase. Decreases the levels of O-GlcNAc transferase. Increases proliferator-activated receptor family of nuclear receptors delta and sirtuin3. Decreases cleaved poly adenosine diphosphate Ribose polymerases and cleaved caspase-3.
**Retinitis Pigmentosa**		
**Authors**	**Study Type and Treatments**	**Results**
Komeima, K., et al., 2006. [2]	rd1 model Mixture of antioxidants: 100 mg/kg LA among others	Mixture of antioxidants: Decreases acrolein and reductions of carbonyl adducts Increases cones density. Improves the function of the retina measured by ERG—mean b-wave amplitude. LA: Increases cones density.
Komeima, K., et al., 2007. [99]	rd10 model and Q344ter model Mixture of antioxidants: 100 mg/kg LA among others	rd10 model: Preservation of cone density. Improves the function of the retina measured by ERG. Slow rod cell death. Q344ter model: Preservation of cone density.
Sanz, M.M., et al., 2007. [3]	rd1 model Mixture of antioxidants: 10 mg/kg LA among others	Mixture of antioxidants: Rescues the number of rows. Decreases avidin and TUNEL positive cells in the ONL.
Miranda, M., et al., 2010. [1]	rd1 model Mixture of antioxidants: 10 mg/kg LA among others	Mixture of antioxidants: Decreases avidin and TUNEL positive cells in the ONL. Increases glutathione peroxidase activity and GSH concentration. Decreases Cyss concentration. No changes in NADPH-diaphorase, neuronal oxide nitric synthase positive cells and nytrotyrosine concentration.
Ramírez-Lamelas, D.T., et al., 2018. [5]	rd1 model LA 100 mg/kg and/or progesterone 100 mg/kg o.a.	Decreases TUNEL-positive cells. Decreases glial fibrillary acidic protein staining. Increases GSH concentration. No changes in glutamate-cysteine ligase catalytic subunit staining.

ERG: electroretinography. GSH: Glutathione. i.p.: Intraperitoneal injection. ONL: Outer nuclear layer. o.a.: Oral administration. ROS: Reactive oxygen species. TUNEL: terminal deoxynucleotidyl transferase dUTP nick end-labeling. VEGF: Vascular endothelial growth factor. ADP: Adenosine diphosphate.

**Table 3:** Antioxidant effects on experimental pathology retinal models.

Antioxidant: Sulforaphane		
**Retinal Pigment Epithelial Cell Damage. Macular Degeneration**		
**Authors**	**Study Type and Treatments**	**Results**
Gao, X., et al., 2001. [107]	ARPE-19 cell line. Oxidative stressors: chemical SFN treatment: 5mM, final concentration with culture medium 0.16–5.0 μM. In vitro study	Cytoprotective effect (cell death reduction). Induction of phase 2 genes.
Gao, X., et al., 2004. [108]	ARPE-19 cell line and mice fibroblast Knockout cells. Oxidative stressors: photooxydation + retinoid SFN treatment: (0–2.5 μM) In vitro study	Antiapoptotic and cytoprotective effects. Induction of phase 2 genes.
Tanito, M., et al., 2006. [109]	BALB/c mice Oxidative stressor: photooxydation SFN treatment: 0.1 or 0.5 mg/d, 5 days, i.p. 0.5/25µL 7 days, o.a. In vivo study K-1034 cell line. Oxidative stressors: Chemical (H_2_O_2_) SFN treatment: 0–10μM (24 or 48 h) 30 μM (6 h). In vitro study	In vivo: Cytoprotective effect and retinal function recovery. Induction of the Trx system. In vitro: SFN does not produce cellular damage (10 μM-48 h). ARE promoter involvement in Trx regulation. Activation of ARE sequence of Trx gene by the Nrf2, small Maf or c-Jun binding proteins.
Zhou, J. et al., 2006. [110]	ARPE-19 cell line. Oxidative stressors: chemical (lipofuscin fluorophore A2E) + photooxidation. (Buthionine sulfoximine treatment: to decrease GSH). SFN treatment: 5-μM sulforaphane for 48 h. In vitro study.	Increased expression and gene activity of enzymes with reductase activity, including GSH, GST, and NQO1.
del V Cano, M., et al., 2008. [111]	ARPE-19 cell line. Oxidative stressors: Chemical stressors SFN treatment: 4 μM (24 h). In vitro study	Regulates the redox ratio and increases cell viability against oxidative stress.
Ye, L. et al., 2013. [112]	ARPE-19 cell line. Oxidative stressors: chemical (H_2_O_2_) SFN treatment:10 μM (12 h) In vitro study	Increase cell viability and antioxidant mechanisms. Identification of several genes induced by SFN, including those related to the response to oxidative stress. An increase of Nrf2 translocation.
Kong, L., et al., 2016. [113]	BALB/cJ mice animal model. Oxidative stressors: photooxydation SFN treatment: 18 mg/kg, 7 days, intracardially. In vivo study.	Retinal protection, to cell and functional level. Increased expression of Trx and Nrf2 and their regulatory elements Ras and ERK. Antiapoptotic effect. Reduction of the mitochondrial intrinsic and caspase-3 antiapoptotic pathways.
O’Mealey, G.B., et al., 2017. [114]	RPE-1 cell line. Cells transfected with siRNA. Apoptotic inducer: staurospine. SFN treatment: 50 μM (2 h). In vitro study.	SFN induces mitochondrial fusion, but independent of the Keap1-Nrf2-ARE pathway. Mechanism: depletion of the fission machinery. Antiapoptotic effect independent of Nrf2.
Dulull, N.K. et al., 2018. [115]	ARPE-19 cell line. Oxidative stressors: chemical (H_2_O_2_). In vitro study	Cytoprotective effect. Induction of phase 2 enzymes (Glutathione-S-Transferase gene). Metabolic effect.
**Retinitis Pigmentosa**		
**Authors**	**Study Type and Treatments**	**Results**
Kang, K., et al., 2017. [118]	Pde6b rd10 animal model, C57/BL6 wild type. SFN: 35 mg/kg, 15 days (from P6 to P20); i.p.	Improvement of retinal function. Reduction of cell degeneration.
**Diabetes Retinopathy**		
**Authors**	**Study Type and Treatments**	**Results**
Maeda, S. et al., S.2014. [122]	Bovine retinal pericytes treated with or without AGE-BSA (Bovine serum albumin). SFN: 0.1 or 0.4 μmol/L sulforaphane.	Reduces the AGE effects on pericytes through the antioxidative mechanisms. Mechanisms: suppression of RAGE expression.
**Retinal Ischemia Reperfusion**		
**Authors**	**Study Type and Treatments**	**Results**
Pan, H., et al., 2014. [123]	Male Sprague-Dawley rats. Animal model by increasing the intraocular pressure SFN: 12.5 mg/kg, 7 consecutive days; i.p. In vivo experiment	Cytoprotective (ganglion and amacrine cells), anti-inflammatory and antioxidant effects. Increase in the Nrf2 nuclear translocation and the HO-1 levels.
Ambrecht, L.A. et al., 2015. [124]	C57BL/6 mice. Animal model by increasing the intraocular pressure. SFN: 25mg/kg, 5 consecutive day; i.p. In vivo experiment	Cytoprotective (retinal morphology) and functional recovery (changes in ERG responses).
**Usher Syndrome (Tubby Model Mice)**		
**Authors**	**Study Type and Treatments**	**Results**
Kong, Li., et al., 2007. [125]	Homozygous tubby mice. C57BL/6J wild type. SFN: 25, 50 or 75 mg/kg. 5 consecutive days (P10–P14) 25 mg/kg from P14 to P34; i.p. Some animals: ERK inhibitor PD98059; i.p.	Cytoprotective effect. Increased Nrf2 retinal level. Reversion of a damaged Trx and TRXR endogenous system. Regulation of the SFN response by external kinases signals.

AGE. Advanced glycation products. ERG: Electroretinography. GSH: Glutathione. GST: Glutathione-S-transferases. HO-1: Heme oxygenase-1. H_2_O_2_: hydrogen peroxide. i.p.: Intraperitoneal injection. NQO1: NAD(P) Quinone reductase. ONL: Outer nuclear layer. o.a.: Oral administration. PGRMC1: Membrane-associated progesterone receptor component 1. RAGE: Receptor for AGE. Ref: References. ROS: Reactive oxygen species. SOD: Superoxide dismutase, TUNEL: Terminal deoxynucleotidyl transferase dUTP nick end labeling. VEGF: Vascular endothelial growth factor.
